# Secernin-1 is a novel phosphorylated tau binding protein that accumulates in Alzheimer’s disease and not in other tauopathies

**DOI:** 10.1186/s40478-019-0848-6

**Published:** 2019-12-03

**Authors:** Geoffrey Pires, Sacha McElligott, Shiron Drusinsky, Glenda Halliday, Marie-Claude Potier, Thomas Wisniewski, Eleanor Drummond

**Affiliations:** 1 Center for Cognitive Neurology and Department of Neurology, New York University School of Medicine, 435 East 30th Street, Rm 1017, New York, NY 10016 USA; 2 Departments of Pathology and Psychiatry, New York University School of Medicine, 435 East 30th Street, Rm 1017, New York, NY 10016 USA; 3 Brain & Mind Centre and Central Clinical School, Faculty of Medicine and Health, University of Sydney, 94 Mallett Street, Camperdown, NSW 2050 Australia; 4 Institut du Cerveau et de la Moelle épinière, CNRS UMR7225, INSERM U1127, UPMC, Hôpital de la Pitié-Salpêtrière, 47 Bd de l’Hôpital, Paris, France

**Keywords:** Alzheimer’s disease, Tauopathies, Phosphorylated tau, Neurofibrillary tangles, Secernin-1, Protein-protein interaction

## Abstract

We recently identified Secernin-1 (SCRN1) as a novel amyloid plaque associated protein using localized proteomics. Immunohistochemistry studies confirmed that SCRN1 was present in plaque-associated dystrophic neurites and also revealed distinct and abundant co-localization with neurofibrillary tangles (NFTs). Little is known about the physiological function of SCRN1 and its role in Alzheimer’s disease (AD) and other neurodegenerative diseases has not been studied. Therefore, we performed a comprehensive study of SCRN1 distribution in neurodegenerative diseases. Immunohistochemistry was used to map SCRN1 accumulation throughout the progression of AD in a cohort of 58 patients with a range of NFT pathology (Abundant NFT, *n* = 21; Moderate NFT, *n* = 22; Low/No NFT, *n* = 15), who were clinically diagnosed as having AD, mild cognitive impairment or normal cognition. SCRN1 accumulation was also examined in two cases with both Frontotemporal Lobar Degeneration (FTLD)-Tau and AD-related neuropathology, cases of Down Syndrome (DS) with AD (*n* = 5), one case of hereditary cerebral hemorrhage with amyloidosis – Dutch type (HCHWA-D) and other non-AD tauopathies including: primary age-related tauopathy (PART, [*n* = 5]), Corticobasal Degeneration (CBD, [n = 5]), Progressive Supranuclear Palsy (PSP, [n = 5]) and Pick’s disease (PiD, [*n* = 4]). Immunohistochemistry showed that SCRN1 was a neuronal protein that abundantly accumulated in NFTs and plaque-associated dystrophic neurites throughout the progression of AD. Quantification of SCRN1 immunohistochemistry confirmed that SCRN1 preferentially accumulated in NFTs in comparison to surrounding non-tangle containing neurons at both early and late stages of AD. Similar results were observed in DS with AD and PART. However, SCRN1 did not co-localize with phosphorylated tau inclusions in CBD, PSP or PiD. Co-immunoprecipitation revealed that SCRN1 interacted with phosphorylated tau in human AD brain tissue. Together, these results suggest that SCRN1 is uniquely associated with tau pathology in AD, DS and PART. As such, SCRN1 has potential as a novel therapeutic target and could serve as a useful biomarker to distinguish AD from other tauopathies.

## Introduction

Alzheimer’s disease (AD) is the most common form of dementia. It is characterized by extracellular aggregation of the amyloid-β (Aβ) peptide into plaques and intraneuronal accumulation of aggregated and hyperphosphorylated tau (pTau) into neurofibrillary tangles (NFTs) [17, 49]. In physiological conditions, tau is a microtubule-associated protein that promotes microtubule stabilization and axonal transport in the brain [6, 74]. In the adult brain, there are 6 tau isoforms derived from alternative splicing of exon 2, 3 and 10 of the MAPT gene [6, 30]. These 6 isoforms differ from one another by the absence or presence of two inserts in the N-terminus and by the presence of either three (3R-tau isoforms) or four (4R-tau isoforms) repeats in the microtubule-binding domain [6, 30, 35]. In neurodegenerative diseases, tau is hyper-phosphorylated and undergoes important conformational changes, causing it to aggregate and form lesions in the brain [3, 31, 32, 35]. In AD, both Aβ and pTau contribute to the development of disease. However, in other neuropathological conditions, dysfunction of tau alone is sufficient to cause dementia [29, 43]. Together, these neurodegenerative diseases are referred to as tauopathies, a group of diseases that includes (but is not limited to) AD, Down Syndrome (DS), Pick’s Disease (PiD), Corticobasal Degeneration (CBD), Progressive Supranuclear Palsy (PSP) and Primary age-related tauopathy (PART) [10, 18, 23]. Each type of tauopathy has a distinct clinical phenotype and distinct neuropathology of pTau aggregates that contain different ratios of 3R and 4R tau isoforms. pTau aggregates in AD, DS and PART contain a mixture of 3R and 4R tau [42], pTau aggregates in CBD and PSP contain only 4R tau [62] and pTau aggregates in PiD contain only 3R tau [43]. It has been proposed that the ratio of 3R:4R tau is of particular pathological importance as this ratio determines the conformation of pTau aggregates and the associated cofactors, which in turn determines mechanism of disease [5, 16, 66].

Protein-protein interactions between pTau and surrounding proteins likely mediates the toxic effects of pTau and influences the development of pTau aggregates in tauopathies. Here, we show that Secernin-1 (SCRN1) is a new protein that interacts with pTau in select tauopathies. We first identified SCRN1 as a new protein associated with AD in our study of the amyloid plaque proteome [11, 12]. In this study, SCRN1 was selected for validation because it was one of the most abundant novel proteins present in amyloid plaques. Our preliminary IHC studies confirmed that SCRN1 was present in amyloid plaques, but unexpectedly, it was present in plaques with a distribution consistent with dystrophic neurites rather than diffuse distribution throughout amyloid plaques. In these preliminary studies we also observed abundant colocalization of SCRN1 in neurofibrillary tangles, suggesting that it may have an important interaction with pTau in AD. Very little is known about SCRN1 and its function is poorly characterized. SCRN1 is a cytosolic 50-kDa protein that was initially identified as a regulator of exocytosis in mast cells [73]. The majority of previous research studying SCRN1 has examined its role in cancer, where it was found to be overexpressed in a variety of cancers and determined to be a tumor-associated protein that correlated with tumor development and poor prognosis [46, 50, 55, 67]. Data mining of transcriptomic and proteomic datasets revealed that SCRN1 is highly expressed in the brain in comparison to other tissue types, both at the mRNA and protein levels [20, 33, 70]. Despite this, we currently have a poor understanding of SCRN1 localization and function in the brain, both in physiological and pathological conditions. SCRN1 overexpression has been observed in the cortex of people with bipolar disorder [59] and in a chick model of retinitis pigmentosa [26]. Recent evidence suggests that SCRN1 may have an important role in regulating endoplasmic reticulum signaling and synaptic vesicle cycling in presynaptic terminals [51], therefore providing new insight into the physiological role of SCRN1 in the brain. Only a few previous studies have described an association between SCRN1 and AD. One study identified SCRN1 as an early marker of neurodegeneration in a transgenic mouse overexpressing human tau [9]. A second study detected SCRN1 in aggregates purified from human AD brain tissue [2]. Recently, SCRN1 was identified as a highly significant CSF biomarker for AD [39]. Together, these three studies strongly hint that SCRN1 may potentially have an important role in AD pathogenesis, perhaps related to synaptic dysfunction, however no studies to date have examined SCRN1 distribution in the human AD brain.

Here, we have performed a comprehensive immunohistochemistry study to characterize SCRN1 throughout the progression of AD and in a range of other neurodegenerative diseases. We show that SCRN1 is a neuronal protein that abundantly accumulates in NFTs and plaque associated dystrophic neurites throughout the progression of AD and in DS and PART. Surprisingly, SCRN1 did not co-localize with pTau-positive glial or neuronal inclusions in CBD, PSP or PiD. Analysis of the interaction of SCRN1 with pTau in AD human brain tissue revealed a significant interaction between SCRN1 and pTau. Together, these results suggest that SCRN1 is uniquely associated with tau pathology in AD, DS and PART. Given its specificity to AD pathology, SCRN1 could serve as a useful AD biomarker and has potential as a novel therapeutic target.

## Methods

### Ethics statement

All cases used in this study were from ethically-approved longitudinally-assessed regional brain donor programs on neurodegenerative diseases. All procedures were performed under protocols approved by the New York University Alzheimer Disease Center, NY, the South Eastern Sydney and Illawarra Local Health District and the Universities of New South Wales and Sydney, Australia. In all cases, written informed consent for research was obtained from the patient or legal guardian, and the material used had appropriate ethical approval for use in this project. All patients’ data and samples were coded and handled according to NIH and NHMRC guidelines to protect patients’ identities.

### Human brain tissue

All human brain samples with a clinical diagnosis of AD, mild cognitive impairment (MCI) or normal aged controls were obtained from the New York University Alzheimer’s Disease Clinical Center (NYU ADC, New York, NY) and were classified neuropathologically using an “ABC” score [56]. This cohort of cases was separated into three groups based on NFT load in the hippocampus and neighboring cortex (“High NFT (**++)**” > 1% NFT load; 0.01%< “Moderate NFT (+)” ≤1% NFT load; “Low/No NFTs (-)” ≤ 0.01% NFT load). The “High NFT” group (*n* = 21) had a clinical diagnosis of AD. The majority of cases included in the “Moderate NFT” group (*n* = 22) had a clinical diagnosis of MCI. All control cases with no cognitive impairment were in the “Low/No NFT” group (*n* = 15). The DS cohort was selected from donated brain tissue collected at the Institute for Basic Research in Developmental Disabilities (IBR, Staten Island, NY) and all had extensive AD neuropathology. The CBD, PSP, PiD and PART cohorts were selected from donated brain tissue held by the University of Sydney. Individual patient information is included below in Table 1.

**Table 1:** Individual case information. Cases were recoded according to their PHF1% load in hippocampus into their respective NFT score (“-“≤ 0.01%; 0.01%< “+” ≤1%;”++” > 1%). Hipp = Hippocampus. BG = Basal Ganglia. TCx = Temporal Cortex. ADRC = Alzheimer’s disease related changes

ID	Diagnosis	ABC score	Sex	Age	NFT score	Region
High NFT 1	AD	A3,B3,C3	M	89	++	Hipp
High NFT 2	AD	A3,B3,C3	F	80	++	Hipp + BG
High NFT 3	AD	A3,B3,C3	F	86	++	Hipp
High NFT 4	AD	A3,B3,C3	F	98	++	Hipp
High NFT 5	AD	A3,B3,C3	F	98	++	Hipp
High NFT 6	AD	A3,B3,C3	F	85	++	Hipp
High NFT 7	AD	A3,B3,C3	F	81	++	Hipp
High NFT 8	AD	A3,B3,C3	F	92	++	Hipp
High NFT 9	AD	A3,B3,C3	F	90	++	Hipp
High NFT 10	AD	A3,B3,C3	F	71	++	Hipp
High NFT 11	AD	A3,B3,C3	M	74	++	Hipp + BG
High NFT 12	AD	A3,B3,C3	F	88	++	Hipp
High NFT 13	AD	A3,B3,C3	M	79	++	Hipp + BG
High NFT 14	AD	A3,B3,C3	F	83	++	Hipp
High NFT 15	AD (early onset)	A3,B3,C3	M	69	++	Hipp
High NFT 16	AD (early onset)	A3,B3,C3	M	63	++	Hipp
High NFT 17	AD (early onset)	A3,B3,C3	M	63	++	Hipp
High NFT 18	AD (early onset)	A3,B3,C3	F	60	++	Hipp
High NFT 19	AD (early onset)	A3,B3,C3	M	62	++	Hipp
High NFT 20	AD (early onset)	A3,B3,C3	F	55	++	Hipp
High NFT 21	AD	A2,B2,C2	M	84	++	Hipp
Moderate NFT 1	AD	A2,B3,C2	M	94	+	Hipp
Moderate NFT 2	AD	A3,B3,C3	F	76	+	Hipp
Moderate NFT 3	AD	A3,B3,C3	M	69	+	Hipp
Moderate NFT 4	AD	A3,B3,C3	M	75	+	Hipp
Moderate NFT 5	AD	A3,B3,C3	F	89	+	Hipp
Moderate NFT 6	MCI	A1,B2,C1	F	97	+	Hipp
Moderate NFT 7	MCI	A1,B2,C1	F	85	+	Hipp
Moderate NFT 8	MCI	A2,B2,C2	M	84	+	Hipp
Moderate NFT 9	AD	A2,B2,C2	F	84	+	Hipp
Moderate NFT 10	MCI	A1,B1,C0	F	84	+	Hipp
Moderate NFT 11	MCI	A1,B1,C1	M	79	+	Hipp
Moderate NFT 12	MCI	A1,B1,C1	M	90	+	Hipp
Moderate NFT 13	MCI	A1,B1,C1	M	74	+	Hipp
Moderate NFT 14	MCI	A1,B1,C0	M	95	+	Hipp
Moderate NFT 15	Normal	A1,B1,C0	M	77	+	Hipp
Moderate NFT 16	MCI	A0,B1,C0	M	88	+	Hipp
Moderate NFT 17	Vascular Dementia	A1,B1,C0	F	89	+	Hipp
Moderate NFT 18	Normal	A1,B1,C0	M	69	+	Hipp
Moderate NFT 19	Normal	A0,B1,C0	F	56	+	Hipp
Moderate NFT 20	Normal	A0,B1,C0	F	59	+	Hipp
Moderate NFT 21	Normal	A0,B1,C0	M	59	+	Hipp
Moderate NFT 22	Normal	A1,B1,C0	F	71	+	Hipp
Low/no NFT 1	Normal	A0,B0,C0	M	77	–	Hipp
Low/no NFT 2	Normal	A0,B0,C0	F	49	–	Hipp
Low/no NFT 3	Normal	A1,B1,C0	M	63	–	Hipp
Low/no NFT 4	Normal	A0,B0,C0	F	51	–	Hipp
Low/no NFT 5	Normal	A0,B0,C0	M	55	–	Hipp
Low/no NFT 6	Normal	A0,B0,C0	M	57	–	Hipp
Low/no NFT 7	Normal	A0,B0,C0	M	59	–	Hipp
Low/no NFT 8	Normal	A0,B0,C0	M	57	–	Hipp
Low/no NFT 9	Normal	A0,B0,C0	M	55	–	Hipp
Low/no NFT 10	Normal	A0,B0,C0	M	54	–	Hipp
Low/no NFT 11	Normal	A0,B0,C0	M	50	–	Hipp
Low/no NFT 12	Normal	A1,B0,C0	M	59	–	Hipp
Low/no NFT 13	Normal	A1,B1,C1	M	89	–	Hipp
Low/no NFT 14	MCI	A2,B1,C1	F	89	–	Hipp
Low/no NFT 15	MCI	A2,B1,C1	M	66	–	Hipp
Down Syndrome 1	DS, AD	A3,B3,C3	F	58	+++	Hipp
Down Syndrome 2	DS, AD	A3,B3,C3	F	59	+++	Hipp
Down Syndrome 3	DS, AD	A3,B3,C3	M	54	+++	Hipp
Down Syndrome 4	DS, AD	A3,B3,C3	M	55	+++	Hipp
Down Syndrome 5	DS, AD	A3,B3,C3	F	54	+++	Hipp
PART 1	PART	A0,B2,C0	M	75	+	Hipp
PART 2	PART	A0,B2,C0	F	86	++	Hipp
PART 3	PART	A0,B2,C0	F	92	+	Hipp
PART 4	PART	A0,B2,C0	M	94	+	Hipp
PART 5	PART	A0,B2,C0	F	90	++	Hipp
PART6	PART	A0,B2,C0	M	92	++	Hipp
CBD 1	CBD	n/a	F	73	+++	BG
CBD 2	CBD	n/a	M	75	+++	BG
CBD 3	CBD	n/a	M	79	++	BG
CBD 4	CBD	n/a	F	80	+	BG
CBD 5	CBD	n/a	M	68	+	BG
PSP 1	PSP	n/a	M	74	+	BG
PSP 2	PSP	n/a	M	71	++	BG
PSP 3	PSP	n/a	M	71	+	BG
PSP 4	PSP	n/a	M	71	+	BG
PSP 5	PSP	n/a	F	87	+	BG
PiD 1	Pick’s Disease	n/a	M	67	+	Hipp
PiD 2	Pick’s Disease	n/a	M	67	++	Hipp
PiD 3	Pick’s Disease	n/a	F	71	+++	Hipp
PiD 4	Picks Disease	n/a	F	65	+++	Hipp
HCHWA-D	Dutch amyloidosis	n/a	F	54	n/a	TCx
FTLD-ADRC 1	CBD with AD	A0,B1,C0	M	72	n/a	Hipp
FTLD-ADRC 2	PSP with AD	A1,B3,C0	F	79	n/a	Hipp

### Immunohistochemistry

Fluorescent immunohistochemistry was performed on formalin-fixed paraffin-embedded tissue sections as described previously [12]. Briefly, 8 μm thick sections were deparaffinized and rehydrated through a series of xylene and ethanol washes. Antigen retrieval was performed by treatment with 88% formic acid for 7 min, followed by boiling in citrate buffer (10 mM sodium citrate, 0.05% Tween-20; pH 6). Sections were blocked with 10% normal goat serum, and incubated overnight at 4 °C with α-SCRN1 primary antibody (1:100; LSBio, catalog #LS-C162903) in combination with the anti-pTau antibodies PHF1 (1:200; generously provided by Dr. Peter Davies, Albert Einstein University, NY, NY), MC1 (1:200; provided by Dr. Peter Davies) or AT8 (1:500; Thermo Fisher Scientific; catalog #MN1020) to label neurofibrillary tangles and dystrophic neurites, RD3 or RD4 antibodies (1:1000; Millipore, catalog #05–803 and #05–804) to label specific 3R or 4R Tau isoforms, or a combination of anti-Aβ antibodies 4G8 (1:4000; BioLegend; catalog #800702) and 6E10 (1:4000; BioLegend; catalog #803001) to label amyloid plaques. Sections were then incubated for 2 h at room temperature with appropriate fluorescent secondary antibodies (all diluted 1:500, from Jackson ImmunoResearch) and coverslipped (ProLong™ Diamond Antifade Mountant, Invitrogen).

### Confirmation of antibody specificity

α-SCRN1 antibody specificity was confirmed by antibody pre-absorption. Two micrograms α-SCRN1 antibody was pre-absorbed by incubation with recombinant human SCRN1 protein (Novus Biologicals, catalog #NBP2–52099) at a ratio of 10:1 for 1 h at RT with over-end rotation. Staining intensity of pre-absorbed antibody was compared to non-preabsorbed α-SCRN1 antibody that was treated in the same way as the preabsorbed antibody and negative control staining that was not treated with primary antibody. Pre-absorption with SCRN1 protein significantly reduced immunostaining, therefore showing evidence of antibody specificity (Additional file 1: Figure S1).

### Quantification of Secernin-1 inside and outside neurofibrillary tangles

Quantification was performed on two separate cohorts. The first cohort included *n* = 58 cases with AD-associated pathology and n = 5 DS cases. Here, fluorescent imaging of the whole brain section was performed at 20x magnification using a NanoZoomer HT2 (Hamamatsu) whole slide scanner using the same imaging settings for all slides. Four pictures containing the cortex and three pictures containing the hippocampus (capturing CA1, CA2, and CA3) were collected at 4x magnification per case for quantification. The second cohort included *n* = 5 cases of CBD, n = 5 cases of PSP, n = 4 cases of PiD, *n* = 6 cases of PART and n = 6 cases of AD selected from the first cohort for comparison. All pictures were collected at 20X magnification per case using a Zeiss LSM700 Confocal microscope. For hippocampal sections (PART, PiD and AD), four pictures containing the hippocampus (capturing dentate gyrus, CA1, CA2 and CA3) and six pictures containing the adjacent entorhinal and temporal cortex were taken for quantification. For basal ganglia sections (PSP, CBD and AD), four pictures of caudate, four pictures of putamen and two pictures of globus pallidus were collected. Quantification was performed using ImageJ. In each cohort, SCRN1 positive staining was identified as all pixels above a binary threshold that was consistent for all images. Multiple image analyses were performed: (1) staining intensity in SCRN1 positive pixels, (2) SCRN1 intensity in pTau positive neurofibrillary tangles or dystrophic neurites, (3) SCRN1 intensity outside neurofibrillary tangles and dystrophic neurites. For SCRN1 intensity in NFTs and dystrophic neurites, NFTs and dystrophic neurites were identified by thresholding the PHF1 image to identify all pixels positive for pTau labelling. A mask of the area selected in this PHF1 image was then applied to the corresponding SCRN1 stained image. The number of SCRN1 positive pixels and average intensity of SCRN1 in positive pixels was quantified inside and outside NFTs and dystrophic neurites.

In our first analysis examining cases with AD-associated pathology, statistical comparison of SCRN1 intensity inside and outside NFTs or dystrophic neurites in the cortex and in the hippocampus in all groups was performed using a one-way ANOVA with Tukey’s multiple comparisons test. Statistical comparison of SCRN1 intensity inside and outside NFTs or dystrophic neurites in other tauopathies (DS, PART, PiD, CBD, PSP) was performed using a two-tailed paired t-test for each group. Representative images for figures (including HCHWA-D) were collected using a Zeiss LSM700 Confocal Microscope. Individual images were collected every 1 μm through the depth of the 8 μm sections at 20x magnification and presented images show the maximum projection image. All images of a particular stain were collected using the same confocal settings.

### Human tissue homogenization

Frozen *post-mortem* frontal cortex tissue from healthy controls (n = 2) and pathologically confirmed AD cases (n = 2) were selected from the same cohort described in Table 1. Grey matter was dissected from each tissue sample and flash frozen until use. Frozen cortical tissue (250 ± 20 mg) was pulverized and dounce homogenized in 5 mL/g (20% w/v) of ice-cold homogenization buffer (50 mM HEPES pH 7.0, 250 mM sucrose, 1 mM EDTA, Protease inhibitor cocktail [cOmplete™ ULTRA Tablets, Mini, EDTA-free; Millipore Sigma; catalog #5892791001]) using approximately 25 pestle strokes. Protein concentration was determined using Bradford protein assay and homogenates were aliquoted and stored at −80 °C until use.

### Co-immunoprecipitation

Immunoprecipitation of SCRN1 was performed using 300 μg of human brain homogenate, and 2 μg of anti-SCRN1 (LSBio; catalog #LS-C162903) or rabbit IgG isotype control (Thermo Fisher Scientific, catalog #02–6102) antibodies. Antibody and brain homogenate were incubated overnight at 4 °C. Immunocomplexes were then incubated with 1.5 mg Dynabeads Protein G magnetic beads (Invitrogen; catalog #1003D) overnight at 4 °C. Beads were washed four times and IP product was eluted in elution buffer (glycine pH 2.8).

### Western blot analysis

Co-IP products and human brain homogenates were analyzed using Western Blot. Samples were mixed in Bolt™ LDS Sample Buffer (Life Technologies) supplemented with 100 mM 1,4-Dithiothreitol (DTT) and boiled 5 min at 95 °C. For pTau western blot, samples were processed without DTT or boiling in order to preserve the oligomeric organization of the paired helical filaments. Proteins were resolved on 12–4% Bis-Tris gels (Life Technologies) and transferred to 0.2 μm nitrocellulose membranes (Bio-Rad). Blots were blocked with 5% milk in TBST for 1 h and probed with primary antibodies at room temperature for 1 h. Western blot results were visualized using enhanced chemiluminescence (Pierce ECL; Thermo Scientific; #32106). Signals were captured using ChemiDoc imaging system (Bio-Rad). The following primary antibodies were used (dilutions): anti-pTau PHF1 (1:200; kindly provided by Dr. P.Davies), anti-Tau Phospho (Ser404, rabbit polyclonal, 1:3000; BioLegend; catalog #SIG-39472), anti-SCRN1 (rabbit polyclonal, 1:1000; LSBio; catalog #LS-C162903), anti-SCRN1 (mouse monoclonal, 1:250; LSBio; catalog #LS-C338451), and anti-GAPDH (1:2000; Cell Signaling; catalog #97166S). Secondary antibodies were anti-rabbit and anti-mouse horseradish peroxidase-labeled antibodies (both 1:3000; GE Healthcare).

## Results

### Secernin-1 distribution in the brain throughout the progression of AD

**Fig. 1:**
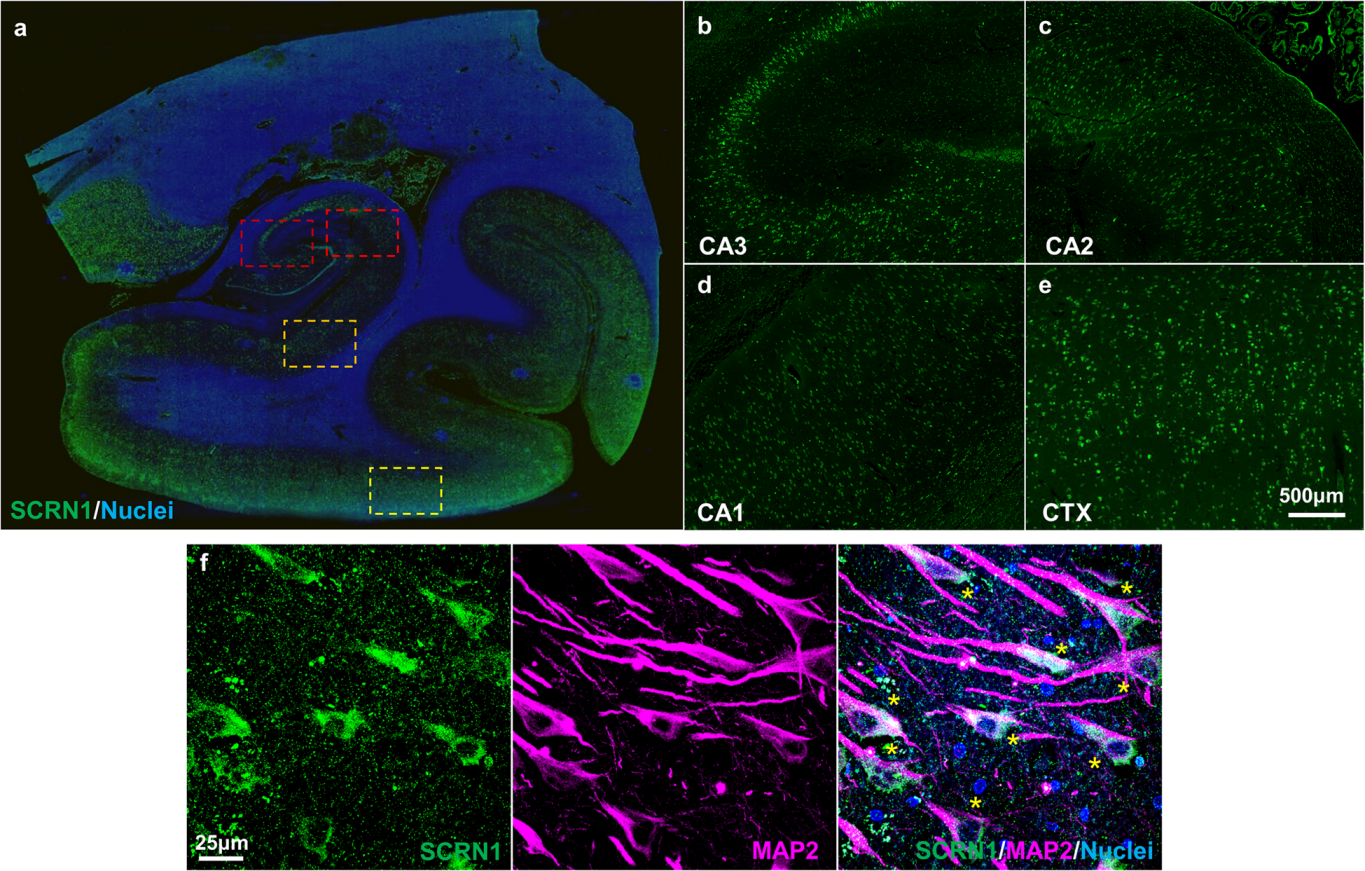
Secernin-1 distribution in the hippocampal section in a cognitively normal subject with no NFTs. **a** Whole-slide fluorescent scan of the hippocampus with α-SCRN1 antibody (green). Boxed regions shown in higher magnification images of CA3 (dark red, **b**), CA2 (red, **c**), CA1 (orange, **d**) and adjacent temporal cortex (yellow, **e**). **f** Double immunohistochemistry of SCRN1 with α-MAP2 antibody showing SCRN1 physiological expression in neurons (asterix) in a cognitively normal subject

In order to determine the physiological localization of SCRN1 and map the accumulation of SCRN1 throughout the progression of AD, we used immunohistochemistry to compare SCRN1 distribution in cases with high AD-associated NFT pathology (*n* = 21 cases), moderate AD-associated NFT pathology (n = 22) and in cognitively normal controls with little or no NFT pathology (n = 15). Cases were included in a specific group according to their NFT load as described in Table 1. SCRN1 was consistently observed in the neuronal cytoplasm in control subjects, confirming that it is a physiological neuronal protein (Fig. 1a-f). The amount of basal SCRN1 neuronal staining in neurons not containing NFTs was similar between the three groups (Fig. 3c, d). There also appeared to be regional differences of basal SCRN1 immunoreactivity: Comparison of SCRN1 in CA1, CA2, CA3 and cortical regions showed comparatively higher SCRN1 staining intensity in CA3, entorhinal cortex and temporal cortex, and lower levels in CA2 and CA1 (Fig. 1). These basal regional differences were observed in all cases regardless of pathology. There was also consistent and bright staining of SCRN1 in the choroid plexus, suggesting that there may be potential secretion, transport or clearance of SCRN1 into or out of the CSF.

**Fig. 2:**
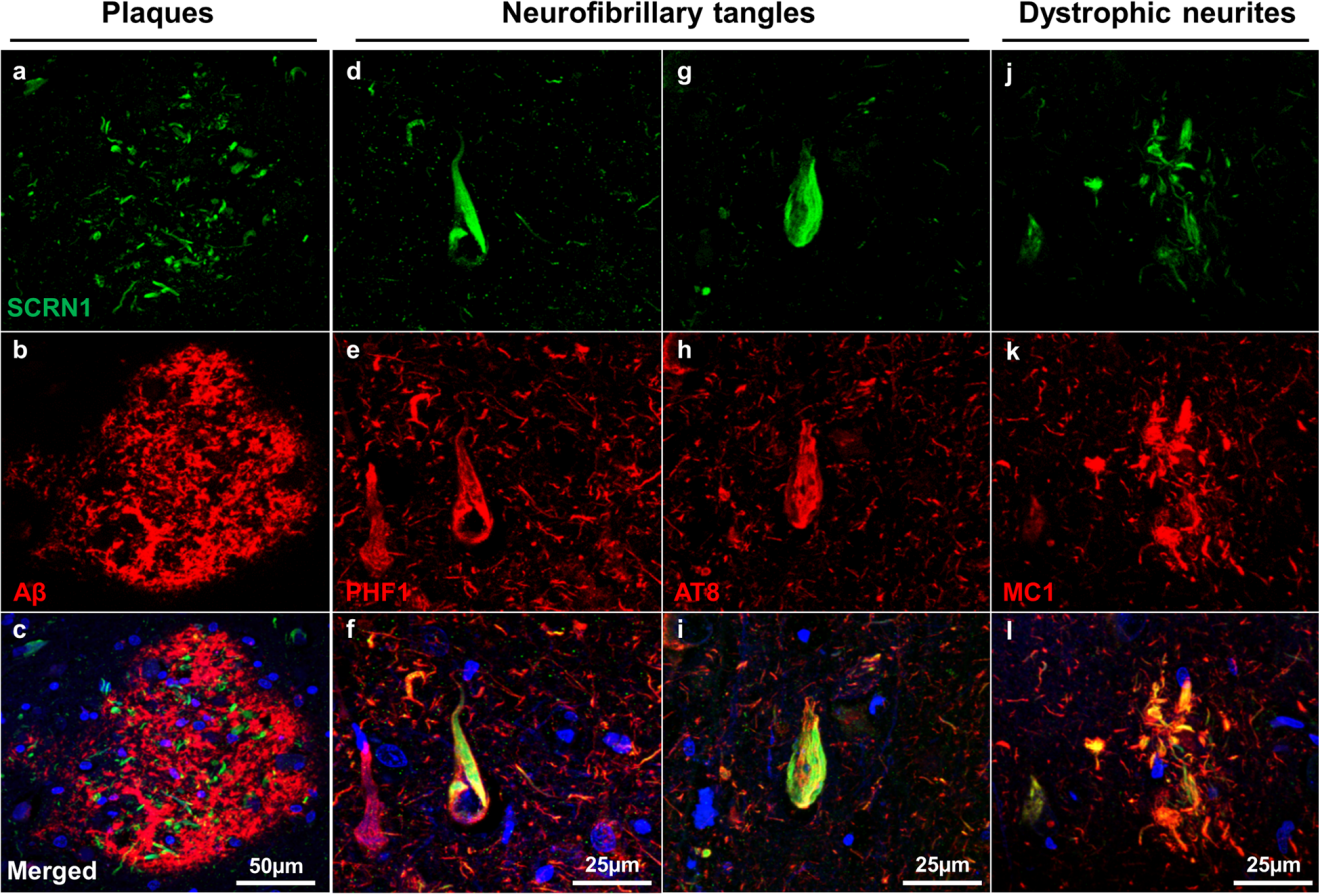
Secernin-1 accumulation in NFTs and dystrophic neurites in AD. Sections were immunostained with α-SCRN1 and 4G8/6E10 (aβ, **a-c**) or the pTau markers PHF1 (**d-f**), AT8 (**g-i**) and MC1 (**j-l**)

A striking pattern of SCRN1 staining was observed in the cases with AD-associated neuropathology. Particularly bright SCRN1 staining was observed in neurofibrillary tangles and in dystrophic neurites present in neuritic plaques (Fig. 2). To determine if there was a particular colocalization of SCRN1 with specific pTau species, we performed double immunohistochemistry of SCRN1 with the antibodies PHF1 (raised against pS404/pS396), AT8 (raised against pSer202/pThr205) and MC1 (conformation-dependent antibody; commonly used as a marker of early-stage tangles). SCRN1 showed consistent colocalization with all three markers, with a striking and obvious accumulation in tangle-bearing neurons and in dystrophic neurites regardless of the pTau species present (Fig. 2). We confirmed that SCRN1 was associated with Aβ plaques, however, the colocalization was limited to the plaque-associated dystrophic neurites rather than the Aβ itself (Fig. 2a-c).

**Fig. 3:**
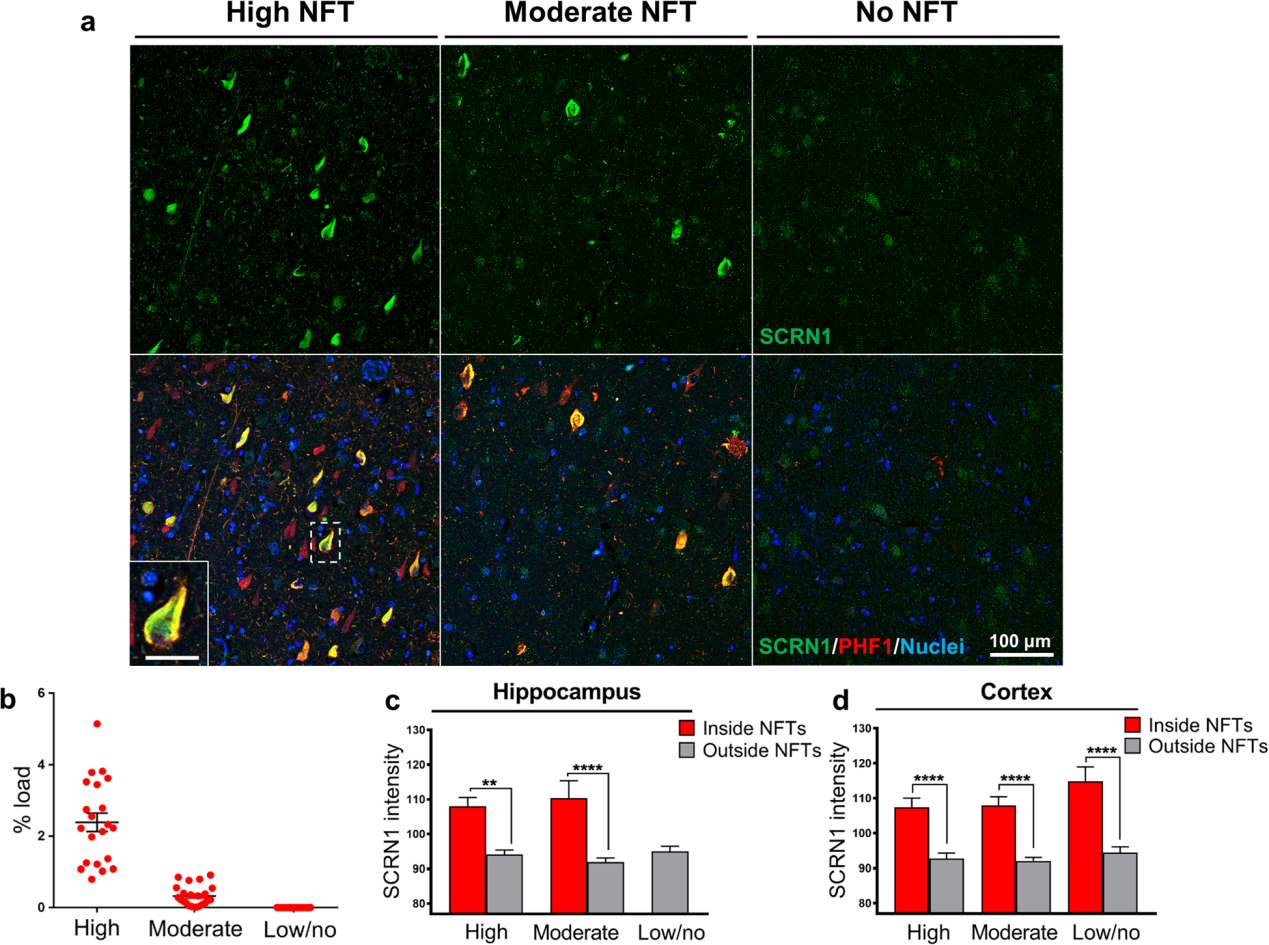
Secernin-1 significantly accumulates in neurofibrillary tangles and dystrophic neurites in AD. **a** Hippocampus sections were immunostained with α-SCRN1 (green) and PHF1 antibody (red) in all three groups. **b** PHF1% load in High, Moderate and Low/no NFTs groups. **c-d** Quantification of staining in the hippocampus and adjacent temporal cortex showed similar basal levels of SCRN1 across the three groups that were not significantly altered by the presence of NFT pathology. In contrast, the amount of SCRN1 in NFTs was significantly higher than that present outside of NFTs in all three groups in hippocampus (**c**) and temporal cortex (**d**). **** p-value < 0.0001; ** p-value < 0.01; one-way ANOVA, Tukey’s multiple comparisons test. Scale bar: 25 μm

In order to determine the timing and extent of SCRN1 accumulation in NFTs we quantified and compared the average staining intensity of SCRN1 inside and outside PHF1-positive NFTs in the cortex and hippocampus in cases with high, moderate and low NFT pathology (Fig. 3a). We found significantly more SCRN1 in NFTs in comparison to surrounding neurons in all cases where NFTs were present (Fig. 3c, d). Importantly, accumulation of SCRN1 was consistently observed in the small number of early-stage NFTs present in the moderate NFT and low/no NFT group (Fig. 3d). This observation, coupled with the colocalization of SCRN1 with the early stage NFT marker MC1, suggests that SCRN1 accumulates early in the formation of NFTs and may therefore have an early role in tangle pathology.

### Secernin-1 does not accumulate in pTau-positive lesions in 3R-tau and 4R-tau specific tauopathies

**Fig. 4:**
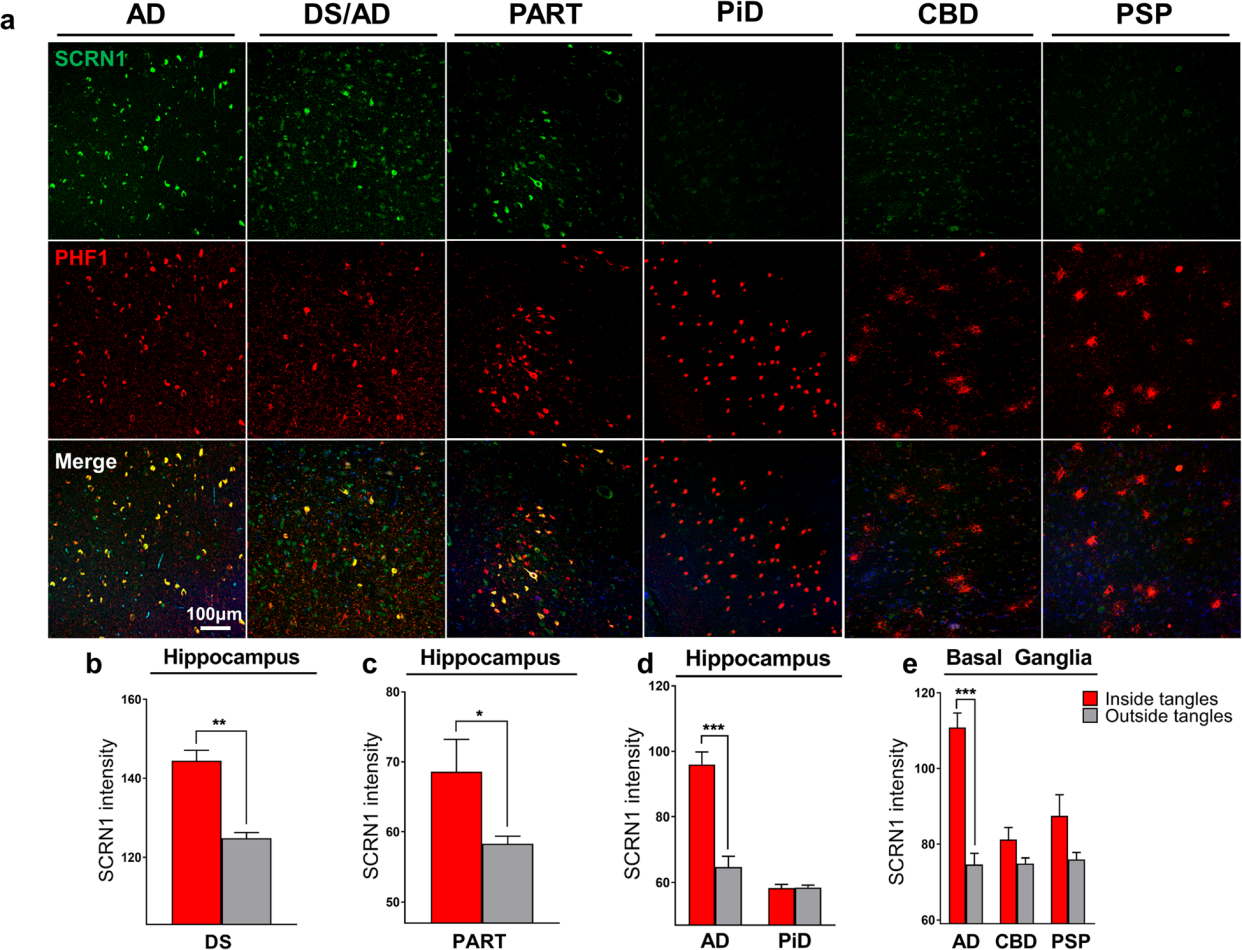
Secernin-1 accumulates in pTau aggregates in AD, DS and PART but not in PiD, CBD and PSP. **a** SCRN1 colocalization with PHF1 in AD, DS and AD, PART, Pick’s disease, CBD and PSP. Representative images show SCRN1 staining in the hippocampus in AD, DS and AD, PART and Pick’s disease and of SCRN1 staining in the basal ganglia in CBD and PSP. SCRN1 (green) accumulated in neurofibrillary tangles and in dystrophic neurites in AD, Down syndrome and PART but not in PHF1 positive aggregates (red) in PiD, CBD or PSP. **b-e** SCRN1 levels were significantly higher in PHF1 positive NFTs and dystrophic neurites than in surrounding neurons in both DS (**b**) and PART (**c**), similar to AD. There was limited/no co-localization of SCRN1 with pTau positive neuropathological lesions in Pick’s disease (**d**), Corticobasal Degeneration and Progressive Supranuclear Palsy (**e**). *** p-value < 0.001, ** p-value < 0.01, * p-value < 0.05; two-tailed paired t-test

**Fig. 5:**
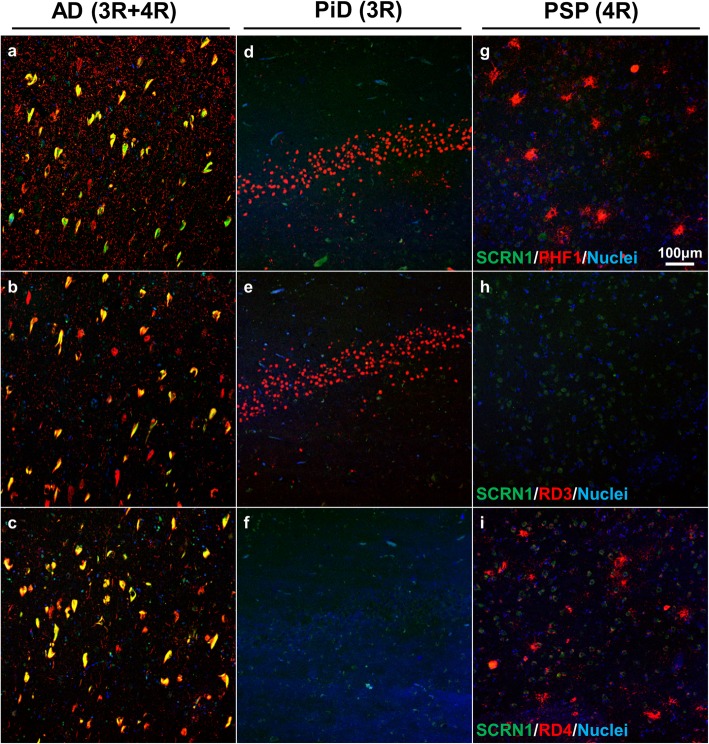
Secernin-1 accumulation is not 3R- or 4R-Tau specific in AD. Sections from hippocampus (**a-f**) or basal ganglia (**g-i**) were immunostained with α-SCRN1 (green) and PHF1 (**a, d, g**; red), RD3 (**b, e, h**; red) or RD4 (**c, f, i**; red) in AD (**a-c**), PiD (**d-f**) or PSP (**g-i**) cases

To address whether SCRN1 also accumulated in NFTs and dystrophic neurites in other tauopathies, we performed double fluorescent immunohistochemistry on basal ganglia or hippocampus sections from cases of CBD (*n* = 5), PSP (n = 5) and PiD (*n* = 4) and compared it to those of AD cases (*n* = 4 of hippocampus, n = 4 of basal ganglia). These tauopathies are known as 4R-Tau (CBD, PSP) or 3R-Tau specific (PiD) tauopathies as the pTau positive lesions present in these diseases exclusively contain either the 4R-Tau or 3R-Tau isoforms. For each disease, we also stained sections with RD3 and RD4 antibodies to confirm the exclusive presence of 4R-Tau in the CBD and PSP cases and of 3R-Tau isoforms in the PiD cases (Fig. 5d-f, g-i). Presence of both 3R and 4R-Tau isoforms was confirmed in the AD cases in both the hippocampus and basal ganglia. In the 4R-Tau specific tauopathies, we observed extensive PHF1 positive staining consistent with the presence of astrocytic plaques (CBD), tufted astrocytes (PSP), pre-tangles, and dystrophic neurites (Fig. 4a) in the basal ganglia. PHF1 pathology in PiD was consistent with Pick’s bodies present in the dentate gyrus, CA regions or entorhinal/transentorhinal region of the cortex (Fig. 4a). Surprisingly, SCRN1 did not accumulate inside any of these pTau immunoreactive lesions in CBD, PSP or PiD (Fig. 4a, Fig. 5d-f, g-i). Quantification of SCRN1 intensity inside and outside PHF1-positive lesions confirmed our findings (Fig. 4d, e), showing that SCRN1 accumulation is a feature unique to NFTs in AD where both 3R-Tau and 4R-Tau isoforms are present and that this occurs across multiple brain regions (hippocampus, cortex and basal ganglia examined in this study). Interestingly, we also observed that SCRN1 consistently colocalized with both 3R or 4R-Tau in NFTs in AD, therefore providing evidence that SCRN1 did not preferentially associate with either 3R or 4R-Tau isoforms (Fig. 5a-c).

### Secernin-1 accumulates in AD-associated NFTs in mixed pathology cases with both FTLD-tau and AD-related neuropathology

**Fig. 6:**
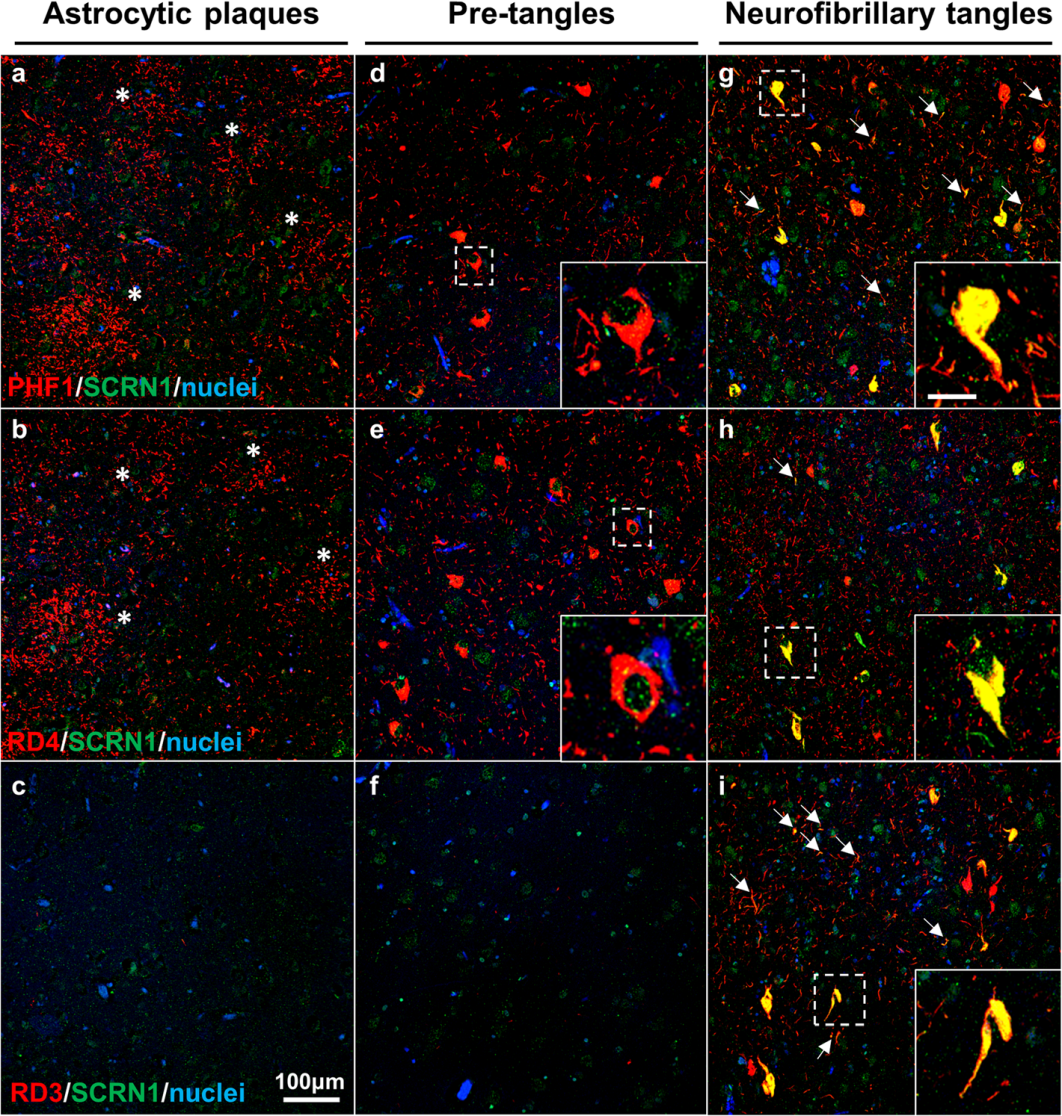
SCRN1 only colocalizes with AD-associated pathology in a CBD case with AD comorbidity. **a-c** Astrocytic plaques (asterix) consistent with CBD pathology contained PHF1 positive pTau, but not SCRN1 (**a**). RD4 (**b**) and RD3 (**c**) immunostaining confirmed 4R tau specificity. **d-f** RD4 positive pre-tangles (**e**) associated with CBD pathology showed immunoreactivity for PHF1 (**d**) but not SCRN1. **g-i** Presence of SCRN1 in lesions containing PHF1(**g**), RD4 (h) and RD3(**i**) positive pTau consistent with AD-associated NFTs pathology and dystrophic neurites (arrows). Scale bar for inserts: 25 μm

**Fig. 7:**
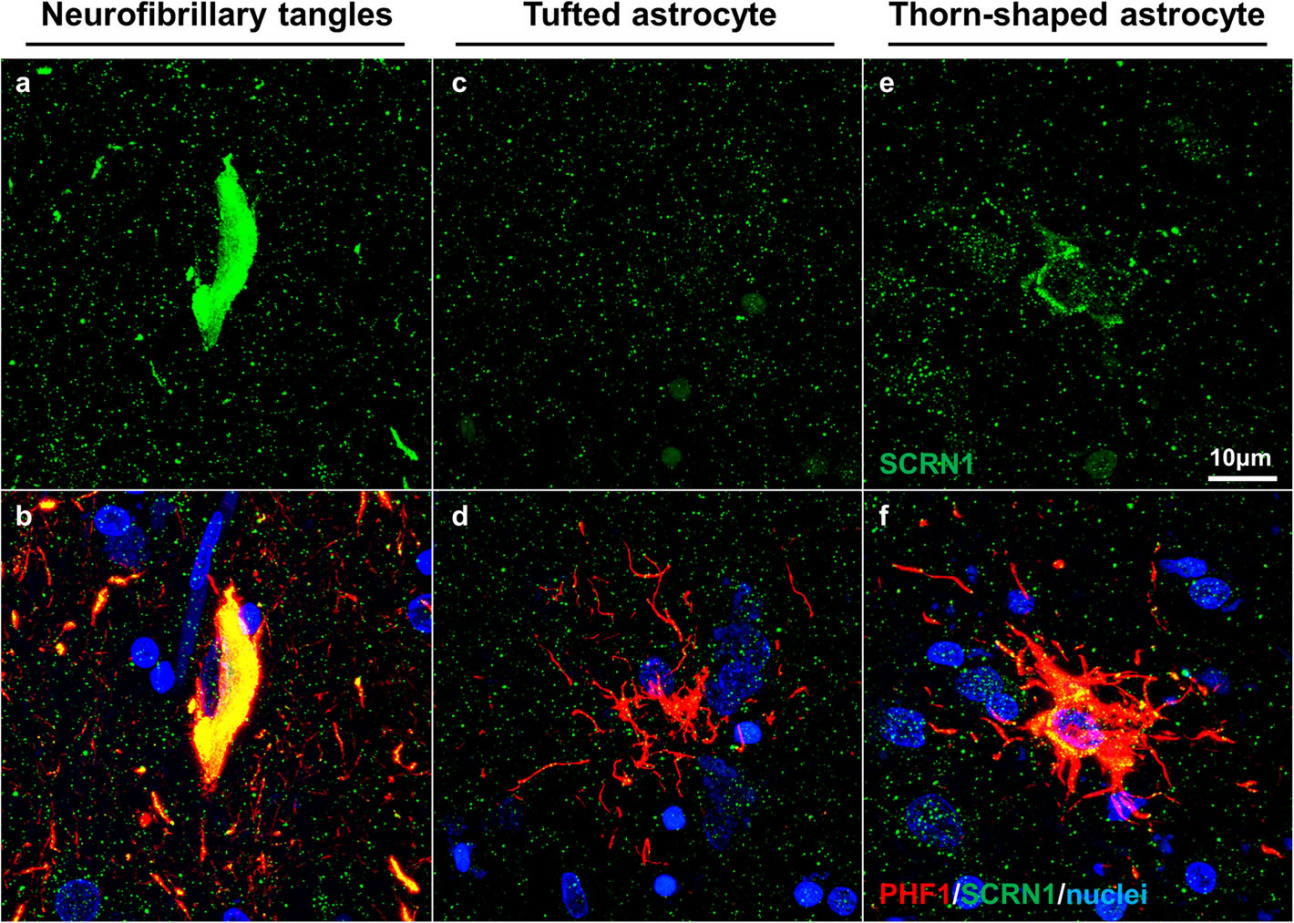
SCRN1 had limited immunoreactivity in ARTAG-associated thorn-shaped astrocytes in a mixed pathology case. **a**, **b** SCRN1 showed prominent accumulation in AD-associated NFTs. **c**, **d** Absence of SCRN1 in PSP-associated tufted astrocyte. **e**, **f** Minimal accumulation of SCRN1 around the nucleus in a thorn-shaped astrocyte

We also examined SCRN1 distribution in two rare cases with mixed pathology: the first subject primarily had CBD-associated neuropathology characterized by astrocytic plaques and pre-tangles, but also had a small number of AD-associated NFTs in the entorhinal cortex and hippocampus. Interestingly, only PHF1 positive lesions with morphology consistent with AD-associated NFTs showed SCRN1 accumulation in this case (Fig. 6g). Given the specificity of 4R tau in lesions present in CBD, we then performed 3R and 4R specific immunohistochemistry on this case of mixed pathology to see if SCRN1 was preferentially present in lesions containing 3R tau. Indeed, this analysis showed striking accumulation of SCRN1 in NFTs containing both 3R and 4R tau (Fig. 6h, i), but not with pre-tangles containing only 4R tau (Fig. 6e). Interestingly, SCRN1 was predominantly present in dystrophic neurites that were immunoreactive for PHF1-positive pTau or 3R tau (Fig. 6g, i) in comparison to those that contained 4R tau (Fig. 6h). The second case of mixed pathology that we examined presented with pTau immunoreactive tufted astrocytes that are commonly observed in PSP, and a small number of thorn-shaped astrocytes that are commonly found in aging-related tau astrogliopathy (ARTAG) [25, 47, 48]. There was also evidence of pre-tangles, as well as some AD-associated NFTs in the hippocampus and adjacent entorhinal cortex. In this case, the most predominant SCRN1 accumulation was again observed in AD-associated NFTs (Fig. 7a, b) and there was no evidence of SCRN1 in tufted astrocytes (Fig. 7c, d). Unexpectedly, we did observe a small amount of SCRN1 accumulation inside thorn-shaped astrocytes (Fig. 7e, f). However in contrast to the widespread SCRN1 colocalization in NFTs, the presence of SCRN1 in thorn-shaped astrocytes was limited to a small proportion of the pTau aggregate that was primarily adjacent to the nucleus (Fig. 7a, b; Fig. 2; Fig. 4a).

### Secernin-1 accumulates in NFTs present in cases of aged Down syndrome and in PART

SCRN1 expression was also examined in cases of DS with AD as well as cases of PART. Individuals with DS all develop AD pathology at an early age [38, 52]. The triplication of the amyloid precursor protein (APP) gene on chromosome 21 results in a progressive accumulation of Aβ starting in early life, such that, by middle age, all people with DS will develop advanced AD pathology. PART is characterized by tau aggregation confined to the entorhinal cortex and hippocampus with no or minimal Aβ deposition [10]. We found that the SCRN1 distribution in DS with AD and PART was similar to that observed in AD; SCRN1 accumulated in NFTs in both types of disease and was present in the dystrophic neurites present in neuritic plaques in DS with AD (Fig. 4a, b, c). These findings suggest that there is a similar mechanism in place for SCRN1 accumulation in AD, DS with AD pathology, and PART.

### Secernin-1 in HCHWA-D

**Fig. 8:**
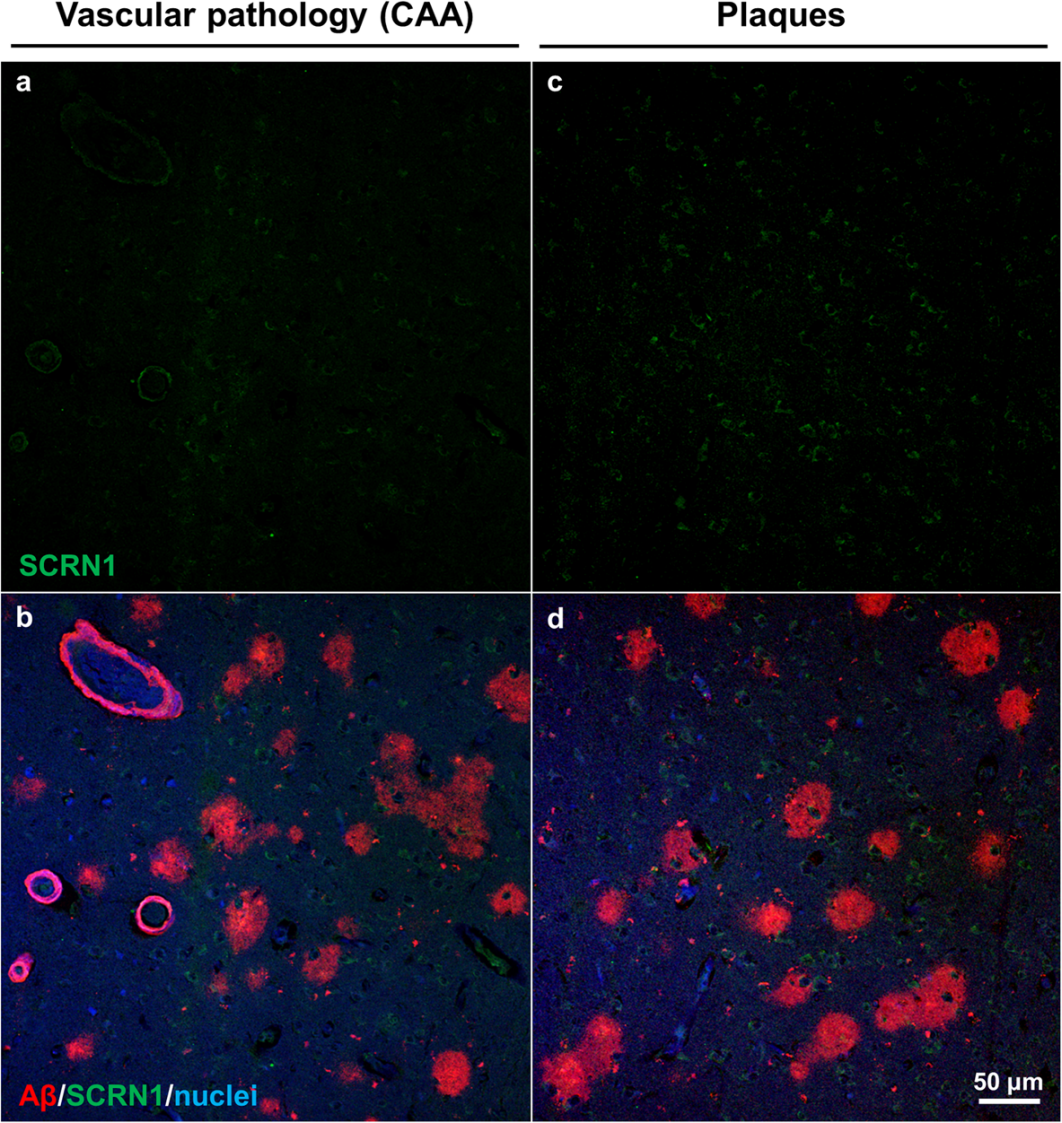
SCRN1 in HCHWA-D. SCRN1/Aβ immunostaining showed no accumulation of SCRN1 in either vascular (**a**, **b**) or parenchymal lesions (**c**, **d**)

We also analyzed SCRN1 distribution in the brain in HCHWA-D, which is a rare autosomal dominant disorder caused by an APP 693 mutation that clinically leads to recurrent hemorrhagic strokes and dementia [4]. The disease is pathologically characterized by CAA and amyloid plaques, without presence of neurofibrillary pathology [4]. In this case, we did not observe any pathologic accumulation of SCRN1, either in vessels or in plaques (Fig. 8).

### Secernin-1 interacts with PHF-tau

**Fig. 9:**
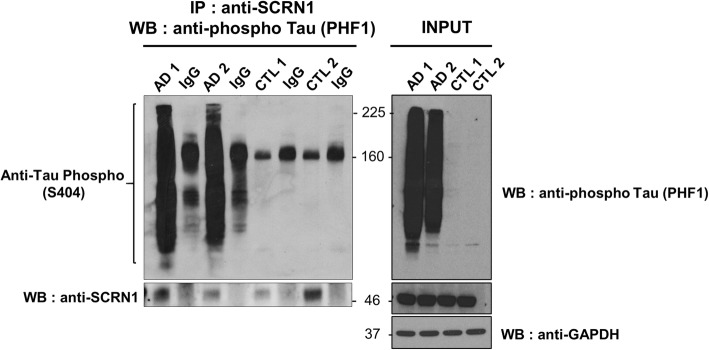
SCRN1 binds to phosphorylated tau in AD cortex tissue. Co-IP was performed on fresh frozen frontal cortex tissue from *n* = 2 AD and n = 2 cognitively normal samples. IP was performed using anti-SCRN1 or rabbit IgG isotype control. SCRN1 pulled down phosphorylated tau in AD cortex samples, but not control cortex samples. Fiftee microgram protein per sample from total homogenate were loaded for inputs. Loading control: GAPDH

The robust colocalization of SCRN1 and pTau observed using immunohistochemistry implies that there may be a direct interaction between SCRN1 and pTau. To test whether this was the case, we performed co-IP of pTau and SCRN1 using fresh-frozen human prefrontal cortex tissue. Immunoprecipitation of SCRN1 from human AD brain homogenates resulted in the co-IP of pTau, implying that SCRN1 interacts with PHF-Tau in vivo (Fig. 9). There was no evidence of any interaction of SCRN1 and pTau in control samples (*n* = 2; Fig. 9).

## Discussion

Here, we present a comprehensive neuropathological study showing that SCRN1 is a novel protein that accumulates specifically in AD, DS and PART, which are 3R/4R tauopathies, but not in other tauopathies that are either 3R or 4R tauopathies. To our knowledge, this is the first example of a protein that distinguishes tau aggregates in different tauopathies. We found that SCRN1 strongly colocalized with NFTs and dystrophic neurites in all brain regions that we examined using AD brain tissue and found SCRN1 to directly interact with phosphorylated tau in AD, a finding that was recently confirmed in an additional study. This study showed significant interaction between SCRN1 and tau in AD, but only minimal interaction in controls, supporting an AD specific interaction between these two proteins [40]. Importantly, SCRN1 was observed in NFTs and dystrophic neurites in preclinical stages of the AD process, suggesting that it could be a key factor associated with the development of NFTs in AD.

The absence of SCRN1 accumulation in pTau lesions present in the 4R-specific tauopathies CBD and PSP raised the possibility that SCRN1 preferentially colocalizes with 3R-tau isoforms, however the lack of SCRN1 accumulation in the 3R-tau specific PiD showed that this was not the case. Adding to the complexity of the relationship between SCRN1 and pTau was the finding that SCRN1 exclusively colocalized with pTau aggregates with NFT morphology and not those aggregates with CBD and PSP-associated morphology in mixed pathology cases. Interestingly, very limited presence of SCRN1 was observed in thorn-shaped astrocytes in one of the mixed pathology cases. Thorn-shaped astrocytes are commonly present in ARTAG, a specific neuropathological condition defined by the existence of pTau-bearing astrocytes in the brain of old-aged individuals [25, 47, 48]. This finding shows that astrocytes have the ability to accumulate SCRN1, suggesting that the absence of SCRN1 in tufted astrocytes or astrocytic plaques is not due to SCRN1 neuronal specificity.

SCRN1 colocalization was also evident in cases of DS with AD and in PART. Importantly, neurofibrillary tangles in DS and PART also contain typical AD-like PHFs that consist of both 3R and 4R tau isoforms [10, 18, 37]. Recent cryo-electron microscopy studies of AD NFTs show that the tau aggregates contain a distinct C-shaped curve with the 3R and 4R tau included in the core of the fibrils [19, 27]. Hence the 3R/4R tau aggregates in AD have a conformation that is distinct from tau species containing only 3R or 4R tau. This was supported by the recent finding that pTau aggregates in AD, PiD and CBD are characterized by different tau molecular structures with different conformations [21, 22, 29, 76]. Together, these findings suggest that the combination of both 3R and 4R tau isoforms in pTau aggregates in AD, DS with AD, and PART could result in the generation of a common tau conformation, or tau “strain”, across these diseases that is then capable of interaction with the same protein co-factors, such as SCRN1. Logically, the different conformation of tau aggregates in other primary tauopathies could prevent interaction with SCRN1. Structure-based inhibitors of tau aggregation are now being developed [64]. Hence targeting the SCRN1/pTau interaction may be a novel therapeutic strategy.

An alternative possibility is that the interaction between SCRN1 and pTau may require the presence of both Aβ and tau. This is supported by the finding that SCRN1 accumulation is present in AD, DS where co-occurrence of Aβ and Tau is observed, while it is not present in tau-only dementias (PSP, CBD, PiD) or in HCHWA-D that only has accumulation of Aβ. The interplay between Aβ and tau is a crucial factor in AD pathogenesis. It has been suggested that this interplay occurs intraneuronally [8, 36, 57, 58], where the interaction with SCRN1 also occurs. One could speculate that Aβ may influence SCRN1 production or activation and therefore influence the subsequent interaction with pTau. Indeed, Aβ is known to stimulate many pathogenic pathways that ultimately influence tau pathology [7, 45, 68, 69, 75]. Therefore, it is possible that the interaction between SCRN1 and pTau is mediated by Aβ in a similar way. However, the abundant presence of SCRN1 in PART does not fit with this hypothesis. Whether PART represents an early stage of AD with very low levels of Aβ or is a distinct pathological entity is still a matter of debate [10, 18]. In our study, we did not find a correlation between the extent of Aβ pathology and accumulation of SCRN1, but rather observed a consistent accumulation of SCRN1 in NFTs in PART. Together, our results suggest that NFTs in PART resemble those present in early stage AD pathology.

The mechanistic involvement of SCRN1 in pTau aggregation and NFT formation is unknown. Further studies are ongoing to determine why SCRN1 is present in NFTs. Domain prediction analysis shows that SCRN1 does not contain a kinase domain, implying that it is unlikely to be involved in the phosphorylation of tau. Interestingly, SCRN1 does contain a conserved 142 amino-acid domain present in members of the Peptidase c69 family. These dipeptidases are cysteine-dependent peptidases that specifically hydrolyze X-Pro motifs [63]. A large number of proteases that cleave tau, such as calpains [24, 61], cathepsins [41, 61, 71], caspases [28, 34, 61, 78] and the lysosomal protease Asparagine endopeptidase (AEP) [61, 77] are also cysteine proteases that are present in intracellular NFTs in AD, similar to SCRN1. The cleavage of tau has been suggested to seed tau aggregation [72]. Therefore, it would be very interesting for future studies to determine if SCRN1 is possibly a novel protease that is involved in pTau proteolysis or degradation.

This study is an example of the powerful and informative nature of unbiased localized proteomics studies, which permit the discovery of novel disease related proteins present within neuropathological features through unbiased analysis [12–15]. It is important to recognize that SCRN1 was only brought to our attention as a novel AD associated protein because of the localized nature of our original proteomics study that identified SCRN1 in dystrophic neurites in amyloid plaques [12]. In contrast, previous transcriptomics and proteomics studies using bulk tissue homogenates did not find it to be one of the top proteins altered in AD [1, 33, 44, 53, 60, 65]. This supports our Western blot results that showed no difference in the amount of SCRN1 in AD and controls in total brain homogenate. The obvious striking accumulation of SCRN1that we observed using IHC shows that localized proteomics studies are necessary and that pathologically important protein differences may be missed in studies of bulk tissue. This is additionally supported by the recent report that examined single-cell transcriptomic changes that found SCRN1 mRNA to be increased in neurons in AD, but not in other cell types [54]. This suggests that the accumulation of SCRN1 in NFTs may be a result of increased localized production of SCRN1 in neurons in AD. Future studies using in situ hybridization on human AD brain sections will help confirm if this increased mRNA production is localized inside NFT containing neurons in AD.

In conclusion, we have shown that the novel protein SCRN1 is associated with neurofibrillary tangles in AD, DS and PART but not in other tauopathies. Future studies both in vitro and in vivo are currently being performed to determine the physiological and pathological function of SCRN1 in the brain, as well as to determine the consequences of its interaction with pTau. Together, our results suggest that SCRN1 is a novel protein that is involved in the pathogenesis of AD.

## Supplementary information

**Additional file 1:** **Figure S1.** Confirmation of α-SCRN1antibody specificity. **a** Western Blot analyses of fresh frozen frontal cortex tissue from *n = 2* AD and *n = 2* cognitively normal samples with PHF1 (pTau ser396/ser404) and two different α-SCRN1 antibodies specifically labelling the 46KDa full-length SCRN1. Immunoblot showed one specific band for SCRN1 and similar SCRN1 levels in AD and cognitively normal samples. Fifteen micrograms protein per sample from total homogenate were loaded. GAPDH was used as loading control. **b** Absorption assay showing the lack of SCRN1 staining after pre-absorption with human recombinant SCRN1 protein. -ve: negative control (no primary antibody).

## Data Availability

The data and material used in this study is available upon request.
