# Supplementary figures and images for: Secernin-1 is a novel phosphorylated tau binding protein that accumulates in Alzheimer’s disease and not in other tauopathies
Source: Acta Neuropathol Commun. 2019 Dec 3;7:195. doi: 10.1186/s40478-019-0848-6 (PMC6892024; doi:10.1186/s40478-019-0848-6)

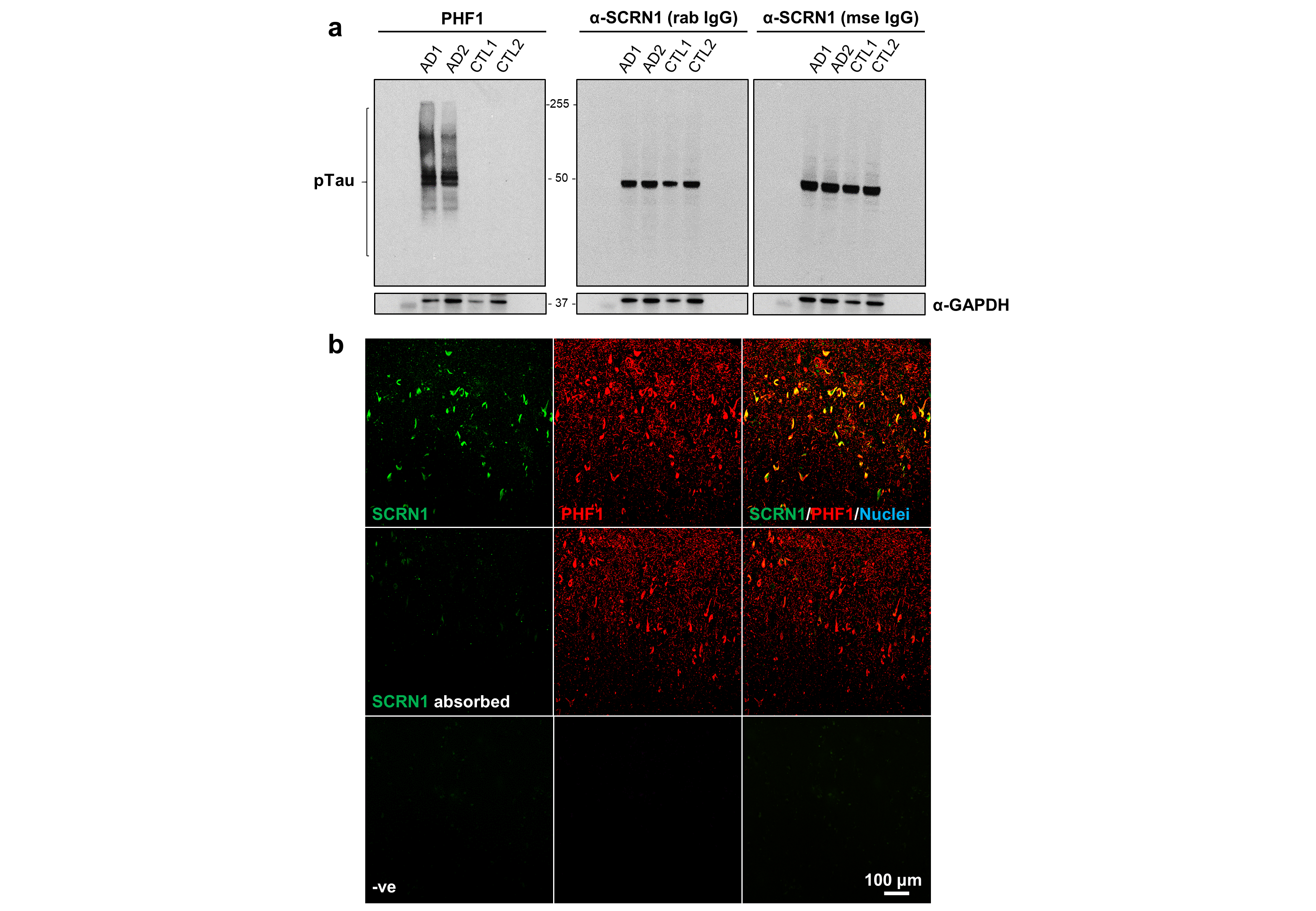

Supplement: Supplementary file 1 — Additional file 1: Figure S1. Confirmation of α-SCRN1antibody specificity. a Western Blot analyses of fresh frozen frontal cortex tissue from n = 2 AD and n = 2 cognitively normal samples with PHF1 (pTau ser396/ser404) and two different α-SCRN1 antibodies specifically labelling the 46KDa full-length SCRN1. Immunoblot showed one specific band for SCRN1 and similar SCRN1 levels in AD and cognitively normal samples. Fifteen micrograms protein per sample from total homogenate were loaded. GAPDH was used as loading control. b Absorption assay showing the lack of SCRN1 staining after pre-absorption with human recombinant SCRN1 protein. -ve: negative control (no primary antibody). [file 40478_2019_848_MOESM1_ESM.tif]
